# Projecting the impact of testing and vaccination on the transmission dynamics of the 2022 monkeypox outbreak in the USA

**DOI:** 10.1093/jtm/taac101

**Published:** 2022-09-01

**Authors:** Qinyue Zheng, Chunbing Bao, Pengfei Li, Annemarie C de Vries, Giulia Giordano, Qiuwei Pan

**Affiliations:** Department of Public Affairs Administration, School of International Affairs and Public Administration, Ocean University of China, Qingdao 266100, China; Department of Information System and Supply Chain Management, School of Management, Shandong University, Jinan 250100, China; Department of Gastroenterology and Hepatology, Erasmus MC-University Medical Center, Rotterdam NL-3015CN, The Netherlands; Department of Gastroenterology and Hepatology, Erasmus MC-University Medical Center, Rotterdam NL-3015CN, The Netherlands; Department of Industrial Engineering, University of Trento, Trento 38100, Italy; Department of Gastroenterology and Hepatology, Erasmus MC-University Medical Center, Rotterdam NL-3015CN, The Netherlands

The USA is emerging as the second epicentre of the 2022 monkeypox outbreak,^1^^,^^2^ in addition to Europe. Delay in diagnosis and reporting is impeding the effective control of this outbreak.^3^ In addition to scaling up and speeding up diagnostic testing, ring vaccination of contacts exposed to confirmed monkeypox cases is being implemented to limit the spread of monkeypox virus (MPXV) in the USA.^4^ This study establishes a mathematical model to specifically recapitulate the transmission dynamics of the current monkeypox outbreak in the USA, and to project the impact of the most urgent interventions focusing on diagnostic testing and ring vaccination.

We constructed an epidemic dynamical model, which partitioned the total US population into seven epidemiological compartments based on the different status of disease diagnosis (Supplementary Figure S1 and Supplementary methods, supplementary data are available at *JTM* online). This model considers heterogeneous (both close/sexual and general) contacts,^2^^,^^5^ associated with different secondary attack rates and different number of contacts exposed to an infected individual. The efficacy of currently available smallpox vaccines against monkeypox was estimated as 85% for both routine vaccination and ring vaccination.^6^ The incorporated parameters and datasets were taken from authentic sources (Supplementary Table S1, supplementary data are available at *JTM* online).

We retrospectively (prior to 15th July) simulated the MPXV epidemic dynamics across the most heavily affected states in the USA. Notable gaps were observed between estimated and confirmed numbers of cases in six representative states. The total number of infected cases is estimated as 1.8 (95% CI 1.3–3.4) times of the reported number (Figure 1A). This suggests that a substantial proportion of infected cases were not diagnosed or reported during this period of the outbreak.

**Figure 1:**
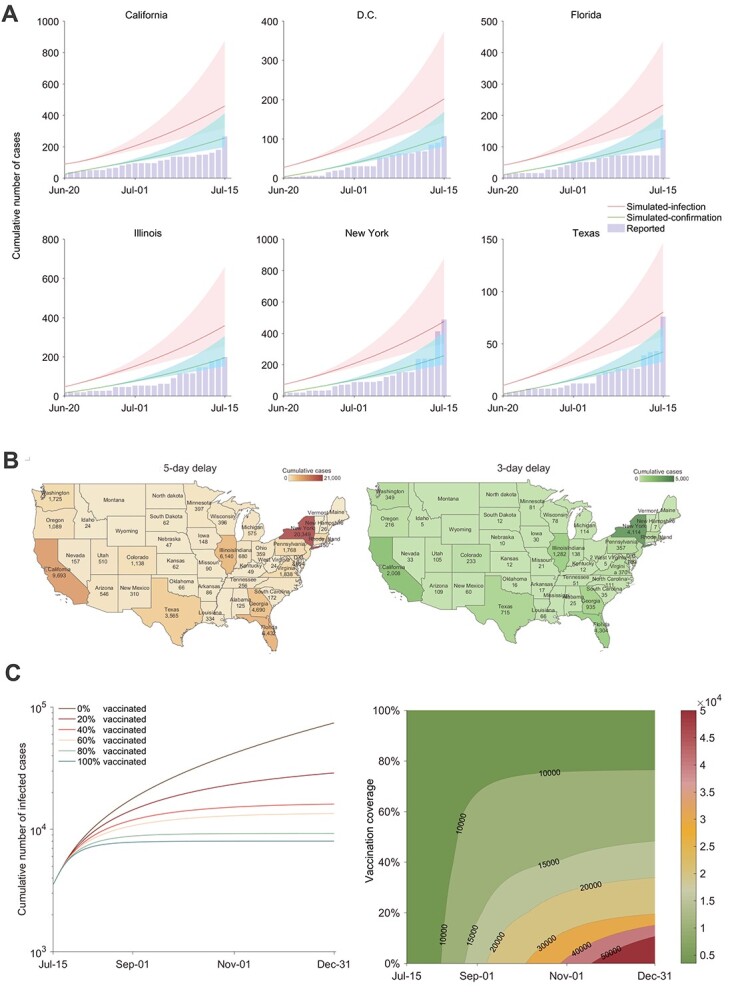
Retrospective simulation of the monkeypox outbreak in the USA, and assessing the impact of rapid diagnosis and ring vaccination on curbing monkeypox spread. (A) Model simulation to fit the spread of MPXV in some representative states of the USA from 20th June to 15th July. The model considers an average delay of 9 days from disease onset to laboratory confirmation in the early outbreak phase. Reported case numbers were obtained from an open-access epidemiological database (https://github.com/globaldothealth/monkeypox). The shaded areas represent the 95% confidence interval related to parameters. (B) We quantified the impact of shortening the delay from disease onset to diagnostic confirmation. On average, a 9-day delay was reported at the early stage of the outbreak. The total number of infections by the end of 2022 was predicted for different states of the USA assuming the confirmation delay can be shortened to 5 or 3 days. (C) When simulating the effectiveness of ring vaccination, the median delay from symptom onset to confirmation was assumed to be 5 days. The efficacy of smallpox vaccines against monkeypox was estimated as 85% for both routine vaccination and ring vaccination.

At the early stage of the outbreak, the average time from disease onset to diagnostic confirmation and reporting required 9 days.^7^ Infected but unconfirmed cases exacerbated the spread of MPXV. If this reporting delay could be shortened to 5 or 3 days, the number of cumulative infections would be reduced by 96.9 and 99.4%, respectively, by the end of 2022 (Figure 1B and Supplementary Figure S2, supplementary data are available at *JTM* online). Notably, 16.2 thousand infections could be prevented for the state of New York alone, by shortening the diagnosis delay from 5 to 3 days.

The implementation of ring vaccination has a clear coverage-dependent effect on curbing the spread of MPXV (Figure 1C). If the median delay from disease onset to confirmation were shortened to 5 days, but without ring vaccination, there would be 74 169 cumulative infections in 2022 in the USA. Vaccinating 20% of exposed contacts would reduce cumulative cases by 32.3% by the third quarter and by 61.1% by the end of 2022. If vaccination coverage reached 40 and 60%, cumulative infections would be reduced by 78.3 and 81.8%, respectively, by the end of 2022. We observed similar effects of ring vaccination across different representative states (Supplementary Figure S3, supplementary data are available at *JTM* online).

As a further demonstration of the robustness of our model, we found that the simulated cumulative infections from 15 July to 15 August, under the scenario of 30% vaccination coverage and 5 days between onset and conformation that is in line with the real-world situation,^2^ are well fitted to the reported data (Supplementary Figure S4, supplementary data are available at *JTM* online). This scenario, when compared with a 9-day delay between onset and conformation, would prevent 29.6% infections.

In summary, this epidemic modelling study recapitulated the early transmission dynamics of the 2022 monkeypox outbreak in the USA. Our model allows us to quantify the importance of shortening the delay from disease onset to diagnostic confirmation and of implementing ring vaccination so as to mitigate the spread of MPXV.

Of note, there are some limitations in this study. First, our model only considers infection numbers, but not the clinical burden related to disease severity, hospitalization or mortality. Second, we did not incorporate the number of sexual partners in transmission, since these numbers remain not available for the US epidemic setting. Third, reinfections and asymptomatic infections were also not considered. Last but not least, once the availability of vaccines increases, follow-up studies will be needed to plan optimal vaccination campaigns that prioritize different high-risk populations.

## Authors’ Contributions

All authors conceptualized and designed the study; Zheng and Bao were involved in acquisition and analysis of data; Zheng, Giordano and Bao took the reponsibility of model building; Pan, Zheng and Bao led the drafting of the manuscript; Giordano, de Vries and Li revised the manuscript; Bao, Zheng, de Vries and Li were responsible for the effectiveness of analysis; Pan, Giordano and Zheng were involved in supervision.

## Conflict of Interest Disclosures

None reported.

## Funding/Support

This work was supported by the National Natural Science Foundation of China (nos. 71901132, 72134004 and 42001121), and the VIDI grant from the Netherlands Organization for Scientific Research (71 901 132).

## Role of the Funder/Sponsor

The funders had no role in study design, data collection and analysis, decision to publish, or preparation of the manuscript.

## Supplementary Material

Supplementary_file_taac101
