# Supplementary material for: Projecting the impact of testing and vaccination on the transmission dynamics of the 2022 monkeypox outbreak in the USA
Source: J Travel Med. 2022 Sep 1;29(8):taac101. doi: 10.1093/jtm/taac101 (PMC9793399; doi:10.1093/jtm/taac101)
Supplement: Supplementary_file_taac101 [file supplementary_file_taac101.pdf]

Supplementary file

Projecting the impact of testing and vaccination on the transmission dynamics of the 2022 monkeypox outbreak in the United States.

Supplementary Methods

Epidemiological dynamical model: Our mathematical model describes the dynamics of the monkeypox disease through seven epidemiological compartments (Figure. 1A). S: susceptible; V: protected by ring vaccination or routine vaccination, cannot be symptomatically infected; E: exposed and latent, but not infectious; I: infected and infectious, with symptoms, undetected; L: infected and infectious, with symptoms, detected (laboratory confirmed); R: recovered; D: dead.

The dynamical system describes the time evolution of the population fractions in the various compartments:

$$\dot{S}_i = -\beta I_i \frac{S_i}{N_i} - \beta' L_i \frac{S_i}{N_i} \tag{1}$$

$$\dot{E}_i = \beta I_i \frac{S_i}{N_i} + \beta' L_i \frac{S_i}{N_i} - \alpha E_i \tag{2}$$

$$\dot{V}_i = \alpha \cdot \upsilon \cdot VE \cdot E_i \tag{3}$$

$$\dot{I}_i = \alpha(1 - \upsilon \cdot VE)E_i - \eta I_i - \gamma I_i - \sigma I_i \tag{4}$$

$$\dot{L}_j = \eta I_i - \gamma L_i - \sigma L_i \tag{5}$$

$$\dot{R}_i = \gamma I_i + \gamma L_i \tag{6}$$

$$\dot{D}_i = \sigma I_i + \sigma L_i \tag{7}$$

where the subscript i represents the state i in the United States. Parameters β and β' are the transmission rates respectively due to undetected and detected infected subpopulations, where β' is 50 times smaller than β thanks to the isolation of detected cases [1 2]. The transmission rate β is estimated by combining the secondary attack rate and the average number of contacts of an infected individual [3]. The secondary attack rates of close contacts and general contacts were defined by [4], and the average number of persons exposed to an infected case was obtained from a surveillance study [5], of which 45% were household or sexual (close) contacts, others were general contacts .

The efficacy of smallpox vaccines against monkeypox is assumed to be 85%. Smallpox vaccines are employed in both routine and ring vaccination campaigns [6 7]. The delay between symptom onset and laboratory testing results is reflected through the diagnosis rate η. It has been reported that the median time between

symptom onset and monkeypox virus testing was 7 days, while the turnaround time from monkeypox virus testing to result availability was 2 days at the early stage of the 2022 outbreak [8]. Therefore, the median confirmation delay in our retrospective simulation was assumed to be 9 days.

Before the outbreak, the total population N_i is assumed to have been composed of susceptible subpopulation (S), constituting 70% of the total population, and vaccinated subpopulation (V), constituting 30% of the total population, to account for individuals born before 1980s who have been routinely vaccinated against smallpox [9] and are therefore protected against monkeypox with 85% vaccine efficacy [7].

Other parameters were also taken from verified sources [6 10].

Scope, timeframe and data sources: The reported monkeypox virus (MPVX) cases were obtained from an open-access database (https://github.com/globaldothealth/monkeypox) compiled from verified sources [11]. We investigated the MPXV epidemiology of the US states. The timeframe of epidemic recapitulation started from June 20 and ended on July 15, 2020. The time window of MPXV spread prediction is six months.

Table S1. Key parameters of the mathematical model.

Parameter	Definition	Value	Source
β	transmission rate due to undetected infections	0.329 (95% CI 0.231-0.429)	[4 5]
β'	transmission rate due to detected infections	0.006	[1 2]
ν	coverage of ring vaccination to exposed individuals	0%	Assumed
VE	vaccine efficacy of ring vaccination	85%	[6 7]
$1/\eta$	delay from symptom onset to diagnosis	9	[8]
$1/\alpha$	incubation period	8.5	[12]
γ	recovery rate	4.54%	[6 10]
d	fatality rate for infected individuals with symptoms	0.22%	[13]

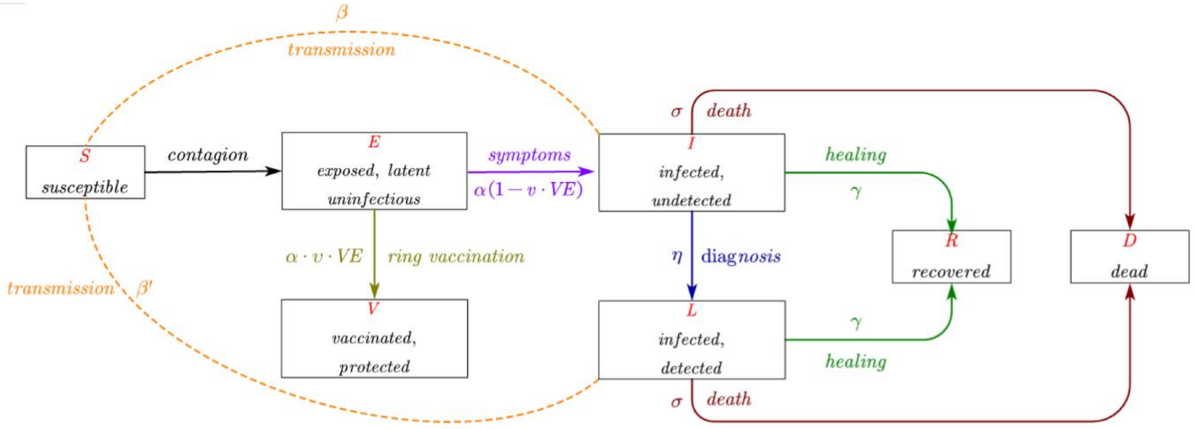

Figure S1. Graphical Scheme of the epidemiological model. In the mathematical model, the vaccinated subpopulation (compartment V) already includes individuals born before 1980s, who received the routine smallpox vaccination. Exposed individuals who are identified and ring vaccinated are transferred from compartment E to compartment V. The transmission rate of detected infections is assumed to be much lower than that of undetected infections, thanks to the isolation of detected cases.

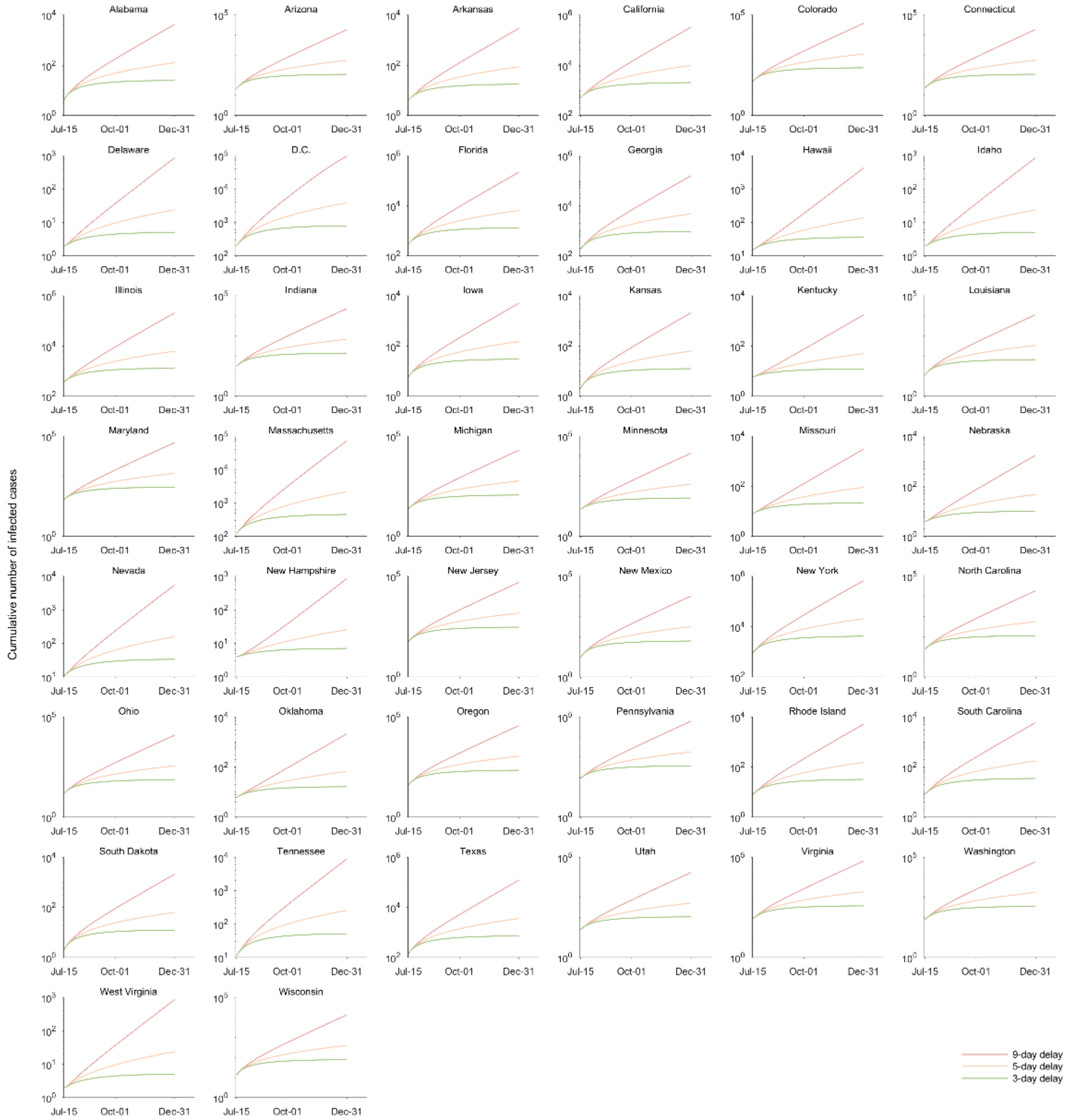

Figure S2. The impact of rapid diagnosis on curbing monkeypox spread in different states of the United States

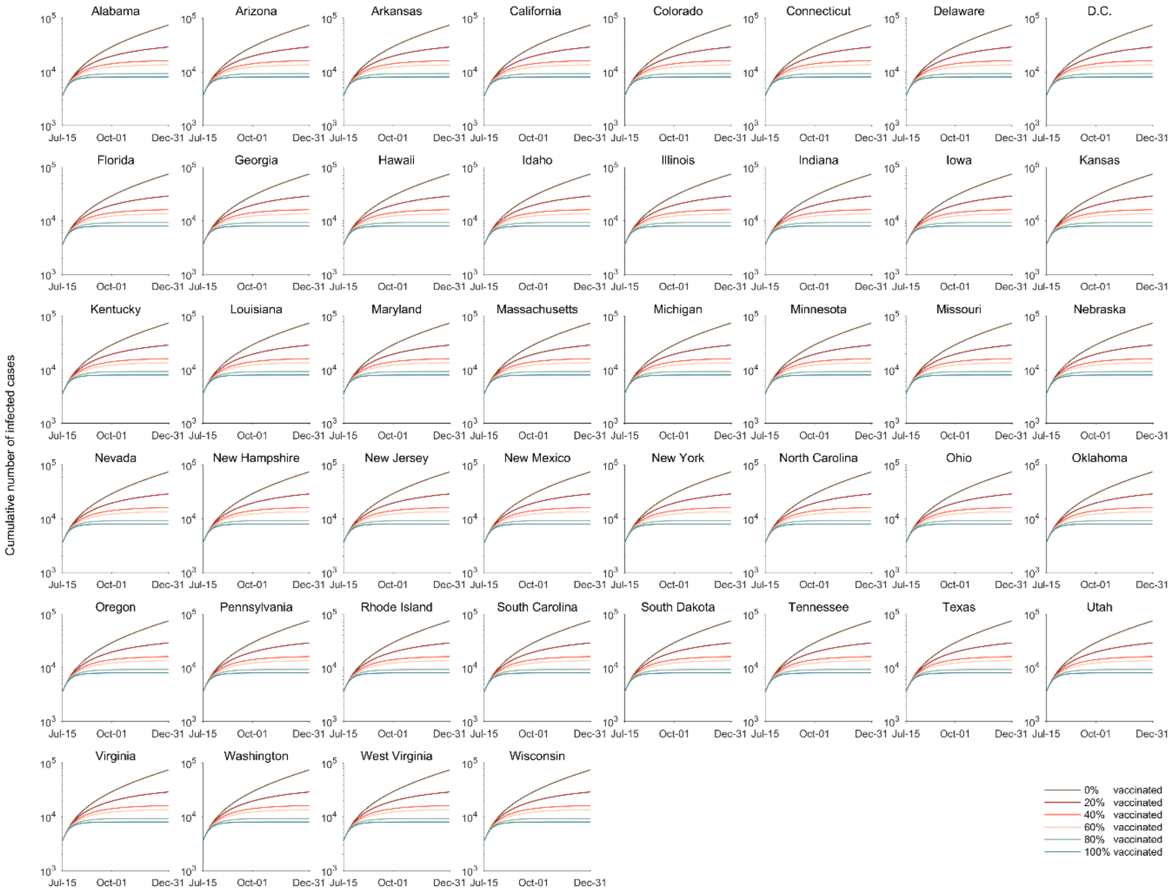

Figure S3. The impact of different coverage of ring vaccination on curbing monkeypox spread in different states of the United States

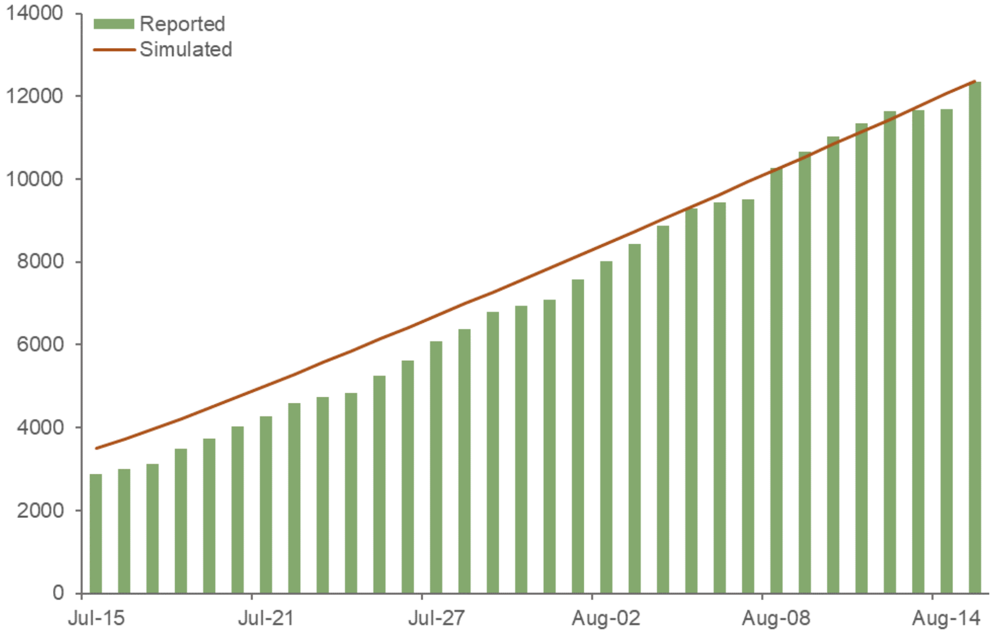

Figure S4. Model simulation to fit the monkeypox epidemic dynamics from 15 July to 15 August in the United States. The total coverage of vaccine of this simulation scenario is 30%, which is close to the aggregated data that 14% of persons reported receipt of pre-exposure smallpox vaccine and 14% of at-risk contacts receive vaccine [5 14]. The delay between onset and conformation was assumed to be 5 days, which is consistent with the observation from the open-access monkeypox database [15]. The reported data were obtained from US CDC [16].

Supplementary references

1. Giordano G, Blanchini F, Bruno R, et al. Modelling the COVID-19 epidemic and implementation of population-wide interventions in Italy. Nature Medicine 2020;**26**(6):1-6 doi: 10.1038/S41591-020-0883-7[published Online First: Epub Date]|.
2. Giordano G, Colaneri M, Di Filippo A, et al. Modeling vaccination rollouts, SARS-CoV-2 variants and the requirement for non-pharmaceutical interventions in Italy. Nature Medicine 2021;**27**(6):993-98 doi: 10.1038/s41591-021-01334-5[published Online First: Epub Date]|.
3. Liu Y, Eggo RM, Kucharski AJ. Secondary attack rate and superspreading events for SARS-CoV-2. The Lancet 2020;**395**(10227):e47 doi: https://doi.org/10.1016/S0140-6736(20)30462-1[published Online First: Epub Date]|.
4. Beer EM, Rao VB. A systematic review of the epidemiology of human monkeypox outbreaks and implications for outbreak strategy. PLoS neglected tropical diseases 2019;**13**(10):e0007791 doi: 10.1371/journal.pntd.0007791[published Online First: Epub Date]|.
5. Vivancos R, Anderson C, Blomquist P, et al. Community transmission of monkeypox in the United Kingdom, April to May 2022. Eurosurveillance 2022;**27**(22):2200422 doi: doi:https://doi.org/10.2807/1560-7917.ES.2022.27.22.2200422[published Online First: Epub Date]|.
6. WHO. Monkeypox. Secondary Monkeypox 2022. https://www.who.int/news-room/fact-sheets/detail/monkeypox.
7. CDC. Monkeypox and Smallpox Vaccine Guidance. Secondary Monkeypox and Smallpox Vaccine Guidance 2022. https://www.cdc.gov/poxvirus/monkeypox/clinicians/smallpox-vaccine.html.
8. Girometti N, Byrne R, Bracchi M, et al. Demographic and clinical characteristics of confirmed human monkeypox virus cases in individuals attending a sexual health centre in London, UK: an observational analysis. The Lancet Infectious Diseases 2022 doi: https://doi.org/10.1016/S1473-3099(22)00411-X[published Online First: Epub Date]|.
9. Simpson K, Heymann D, Brown CS, et al. Human monkeypox – After 40 years, an unintended consequence of smallpox eradication. Vaccine 2020;**38**(33):5077-81 doi: https://doi.org/10.1016/j.vaccine.2020.04.062[published Online First: Epub Date]|.
10. CDC. Monkeypox. Signs and Symptoms. Secondary Monkeypox. Signs and Symptoms 2022. https://www.cdc.gov/poxvirus/monkeypox/symptoms.html.
11. Kraemer MUG, Tegally H, Pigott DM, et al. Tracking the 2022 monkeypox outbreak with epidemiological data in real-time. The Lancet Infectious Diseases 2022;**22**(7):941-42 doi: 10.1016/S1473-3099(22)00359-0[published Online First: Epub Date]|.
12. Miura F, van Ewijk CE, Backer JA, et al. Estimated incubation period for monkeypox cases confirmed in the Netherlands, May 2022. Eurosurveillance 2022;**27**(24):2200448 doi: doi:https://doi.org/10.2807/1560-7917.ES.2022.27.24.2200448[published Online First: Epub Date]|.
13. Kozlov M. Monkeypox vaccination begins — can the global outbreaks be contained? Secondary Monkeypox vaccination begins — can the global outbreaks be contained? 2022. https://www.nature.com/articles/d41586-022-01587-1.
14. CDC. Epidemiologic and Clinical Characteristics of Monkeypox Cases — United States, May 17–July 22, 2022. Secondary Epidemiologic and Clinical Characteristics of Monkeypox Cases — United States, May 17–July 22, 2022 2022. https://www.cdc.gov/mmwr/volumes/71/wr/mm7132e3.htm.
15. Global.health team. Monkeypox 2022 repository. Secondary Monkeypox 2022 repository 03 July, 2022 2022. https://github.com/globaldothealth/monkeypox.
16. CDC. U.S. Monkeypox Case Trends Reported to CDC. Secondary U.S. Monkeypox Case Trends Reported to CDC 2022. https://www.cdc.gov/poxvirus/monkeypox/response/2022/mpx-trends.html.
